# Molecular alignment in degenerated dissociation channels in strong laser fields

**DOI:** 10.1038/s41598-017-02738-5

**Published:** 2017-05-31

**Authors:** Wei Lai, Alan Heins, Chunlei Guo

**Affiliations:** The Institute of Optics, University of Rochester, Rochester, New York 14627 USA

## Abstract

In this work, we study strong-field molecular alignment in, for the first time, degenerated channels following the same charged states of molecules. By measuring the angular distribution of dissociation fragments from two degenerated N^+^ + N^+^ channels of N_2_, we observe an opposite angular distribution development in these two channels, one expanding and one contracting, when the laser intensity increases. Our further study shows that the expanding channel comes from a nonsequential transition, while the contracting channel involves a sequential transition. We also study the time sequence of the sequential and nonsequential transitions and find that the opposite angular distribution development is due to the different degrees of molecular alignment in these two degenerated channels.

## Introduction

In strong laser fields, small molecules can be rapidly aligned with the laser polarization due to the torque on the laser-induced molecular dipole moment^1–5^. Molecular alignment can be realized either adiabatically or non-adiabatically, when the laser pulse duration is longer or shorter than the rotational period of the molecule, respectively^5^. The non-adiabatic alignment by using ultrashort laser pulses is of particular interest since the aligned molecules are under field-free conditions and therefore subsequent dynamics will not be influenced by external fields^5^. Molecular alignment has been an active research topic in strong field science in the past two decades, due to its potential applications in both physics including ultrafast dynamic imaging^4, 6–9^, molecular tomography^10–12^, and attosecond science^5, 13–15^, and chemistry, since most chemical reactions depend on the relative orientation of the reactants and the absorption of polarized light in photochemical processes also depends on the alignment of the molecule^2, 5, 10, 16^.

For molecular dissociative ionization, various dissociation channels following different charged states can occur due to the multiple degrees of freedom and the complex multielectron effects of molecules^17–21^. Furthermore, degenerated channels following the same charged state have also been observed in strong field-molecular interactions^22–24^. It is of great importance to study the alignment effect in these different dissociation channels of a molecule, since different dissociation channels usually lead to different end products with different kinetic energy and/or residing in different excited states^17–21^. If the end products can be differentiated or controlled by the alignment effect, it may open up a possibility to control chemical pathways simply with laser alignment effect^2, 5, 10, 16^. Molecular alignment in dissociative ionization channels has been widely studied in the past^1–12^; however, no studies have been performed on degenerated channels from the same charge states in a molecule.

Our previous studies identified two degenerated N^+^ + N^+^ channels from N_2_, a fast channel with a higher kinetic energy release (KER) and a slow channel with a lower KER^22, 23, 25^. In this work, we use these two channels to study, for the first time, strong-field induced molecular alignment effect in degenerated dissociation channels. When we measure the angular distribution of the dissociation fragments as increasing the laser intensity, an opposite angular distribution development is observed in these two channels, one expanding and one contracting. We identify that the expanding angular distribution comes from the fast channel involving a nonsequential transition, whereas the contracting angular distribution comes from the slow channel that involves a sequential transition. Our further study on the time sequence of the sequential and nonsequential transitions indicate that the opposite angular distribution development is due to different degrees of strong-filed induced molecular alignment in these two degenerated channels.

## Results

### Polar charts of molecular orientation distribution

**Figure 1:**
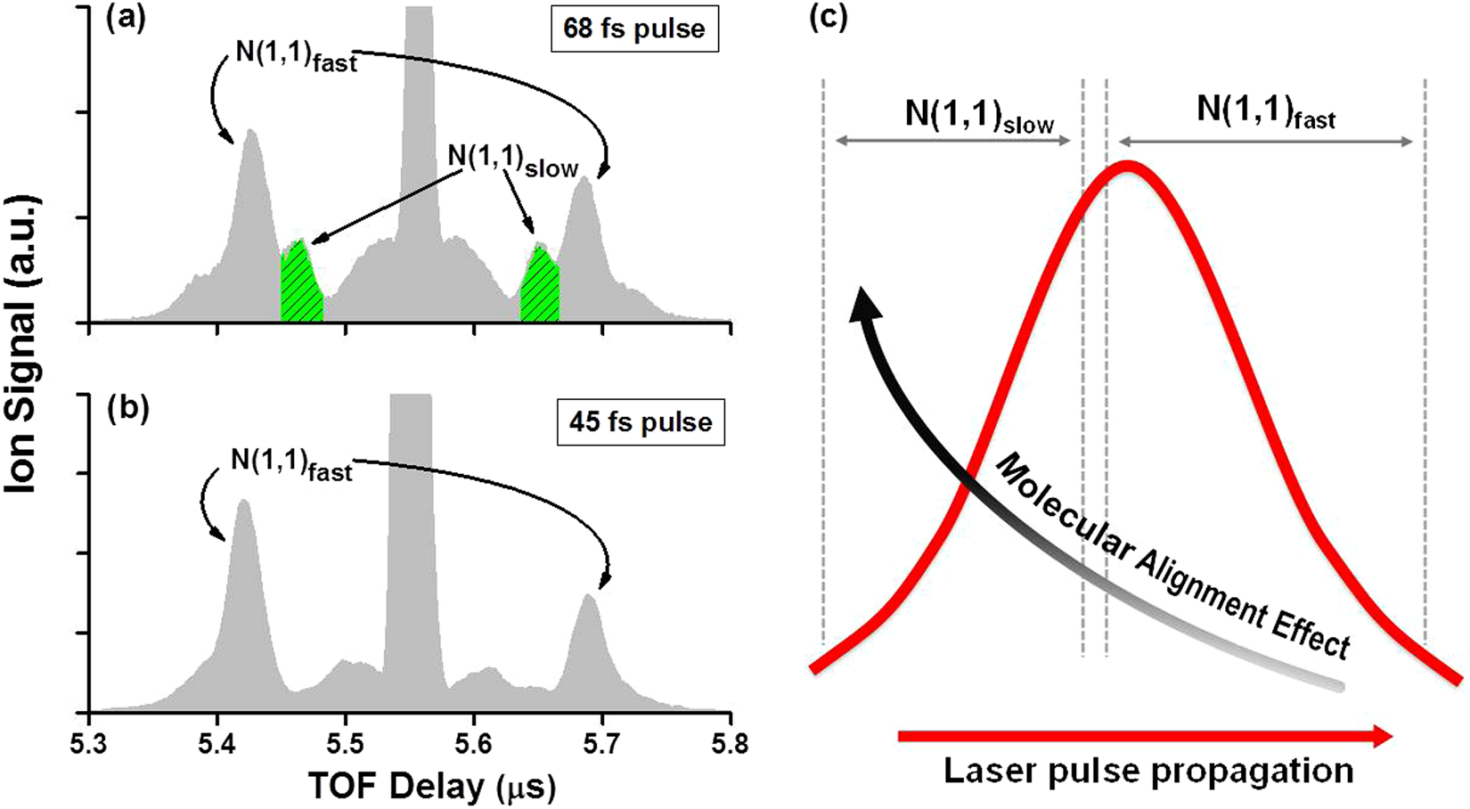
TOF spectra for the N(1,1)_fast_ and N(1,1)_slow_ channels from ionization and dissociation of N_2_ using linearly polarized (**a**) 68-fs and (**b**) 45-fs pulses. N(1,1)_slow_ is clearly seen in 68-fs pulses but nearly disappears in 45-fs pulses. (**c**) Schematic illustration of the formation time sequence of N(1,1)_fast_ and N(1,1)_slow_.

**Figure 2:**
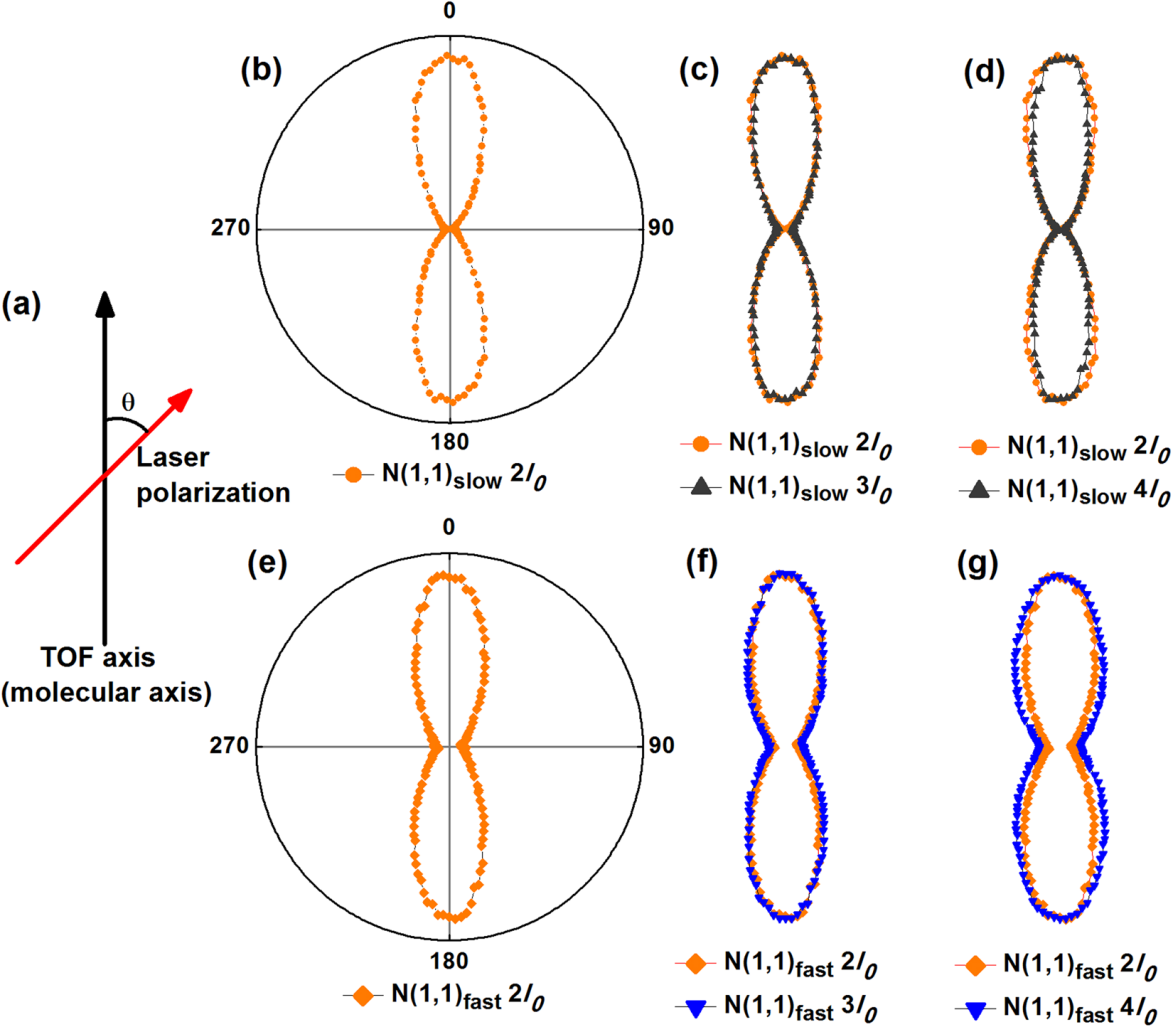
Polar charts of measured angular distribution of N(1,1)_slow_ [(**b**–**d**)] and N(1,1)_fast_ [(**e**–**g**)] at different laser intensities: 2*I*_*0*_, 3*I*_*0*_, and 4*I*_*0*_, where *I*_*0*_ = 10^14^ W/cm^2^. The magnitude of the data points is normalized to fit the window and for easy comparison by overlapping with other distribution profile, and therefore is in arbitrary units. The profile at 2*I*_*0*_ is superimposed in each of the other profiles at 3*I*_*0*_ and 4*I*_*0*_ for each channel. The lower half of the data in each panel is mirrored from the upper half. (**a**) Illustration of the geometric configuration of the experimental setup. The TOF axis (i.e. the molecular axis) is vertical in this plane while the direction of laser polarization varies at angle θ with respect to the TOF axis in the same plane.

The two degenerated N^+^ + N^+^ channels from double-ionization-induced dissociation of N_2_ have been identified in our previous studies^22, 23^: a fast N^+^ + N^+^ channel with a higher KER of 7.0 eV [labeled as N(1,1)_fast_], and a slow N^+^ + N^+^ channel with a lower KER of 3.8 eV [labeled as N(1,1)_slow_] (see Fig. 1(a), which shows the TOF mass spectrum of N^+^ ion peaks obtained with linearly polarized 68-fs pulse at an intensity of 4 × 10^14^ W/cm^2^). Figure 2 shows the polar charts of the measured molecular orientation distribution of N(1,1)_slow_ and N(1,1)_fast_ obtained with 68-fs pulses at different laser intensities, 2*I*_*0*_, 3*I*_*0*_, and 4*I*_*0*_, with *I*_*0*_ = 10^14^ W/cm^2^. To see the change of the distribution profile, we superimpose the curve at 2*I*_*0*_ to all other curves, shown as the solid dots in Fig. 2. Distinctive difference can be seen between the two channels when the angular distribution profile develops with laser intensity. For N(1,1)_slow_ [Fig. 2(b),(c) and (d)], the profile at 4*I*_*0*_ clearly gets contracted compared to the 2*I*_*0*_ guideline, which can be seen quantitatively from the <cos^2^θ> measure of alignment: 0.83 at 2*I*_*0*_ and 0.86 at 4*I*_*0*_. A contraction in the angular distribution profile with increasing intensity, i.e. an increase in the <cos^2^θ> measure, indicates that molecules are more aligned towards the laser polarization^5, 6, 11, 26^. Therefore, as the laser intensity increases from 2*I*_*0*_ to 4*I*_*0*_, more molecules are aligned towards the laser polarization when N(1,1)_slow_ is created. In contrast, for N(1,1)_fast_ [Fig. 2(e),(f) and (g)], an expansion is clearly seen in the angular distribution profile when the laser intensity increases from 2*I*_*0*_ to 4*I*_*0*_, with the <cos^2^θ> measure decreasing from 0.77 to 0.73. While an expansion in the angular distribution profile with increasing intensity has not been commonly seen in previous molecular alignment studies, nor explicit mechanisms have been established^5, 6^, it certainly does not indicate a higher degree of alignment in this channel.

Note that an isotropic distribution across all θ corresponds to a <cos^2^θ> value of 0.33. However, due to the detection setup of the experiment, the signal measured is not across the entire space but rather is a slice of the entire spatial distribution. For this reason, an isotropic distribution gives a measure of <cos^2^θ> to be 0.5. At the low intensity end of our experiments, both N(1,1)_slow_ and N(1,1)_fast_ show a <cos^2^θ> value larger than 0.5 and this could be due to two possible mechanisms: the ionization rate of the channel is not angularly constant, and/or a certain degree of alignment that has been achieved when the channel is formed^5, 12^. According to previous studies, the angular ionization rate of a molecular fragmentation channel, which is closely related to the molecular orbital characteristics, could significantly impact the angular distribution of fragments^6, 11, 12, 27–29^. In the following studies, we will explore which effect plays a major role leading to the large <cos^2^θ> value in these two channels.

### The relationship of the laser intensity, molecular ionization rate, and the angular distribution data

**Figure 3:**
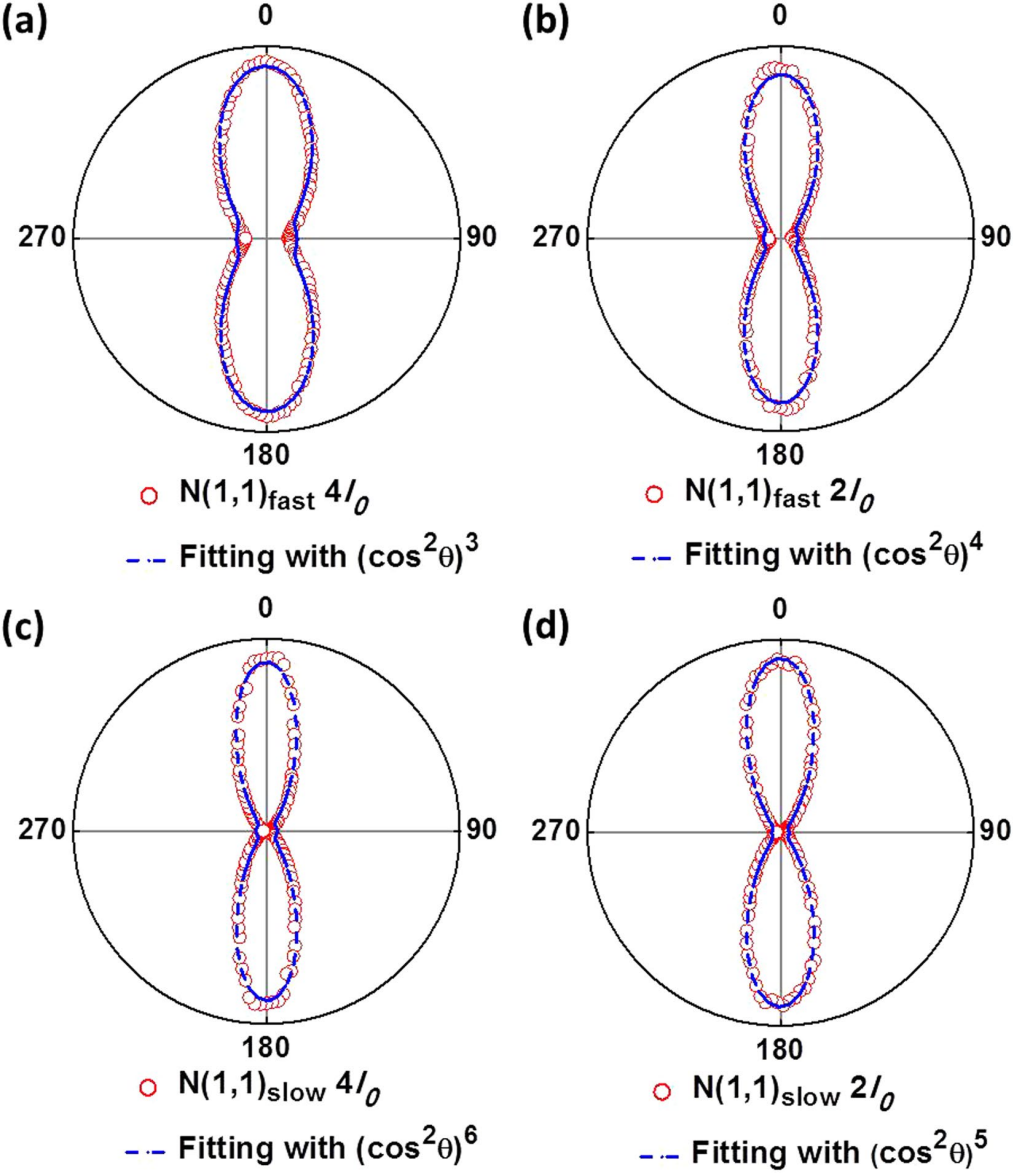
(**a**–**d**) Fitting of the angular distribution data with a cos^2^θ function. See text for more details.

When we rotate the laser polarization and introduce an angle *θ* between the laser polarization and the TOF axis (i.e. the molecular axis since only ions having their velocity aligned with the TOF axis will be detected), the equivalent *E*-field strength along the molecular axis is *Ecosθ* and the equivalent laser intensity along the molecular axis is in proportion to (*Ecosθ*)^2^. When we vary the angle *θ*, we vary the equivalent laser intensity proportion along the molecular axis and thus vary the corresponding ion yield rate. Therefore, we fit the angular distribution data by using a *cos* ^2^ *θ* function with an exponential order n, i.e. (*cos* ^2^ *θ*)^n^, aiming at revealing the correlation between the intensity dependence of the angular distribution data and the intensity dependence of the ion yield rate of the channel. The fitting is shown in Fig. 3(a–d). We can see that N(1,1)_fast_ is fitted by (*cos* ^2^ *θ*)^3^ at the high intensity end and (*cos* ^2^ *θ*)^4^ at the low intensity end (plus an angle-independent offset that accounts for the weak signal of N(1,1)_fast_ when the laser polarization is perpendicular to the molecular axis, i.e. the components at *θ* = 90 and 180 degree in Fig. 2), while N(1,1)_slow_ is fitted by (*cos* ^2^ *θ*)^6^ at the high intensity end and (*cos* ^2^ *θ*)^5^ at the low intensity end.

**Figure 4:**
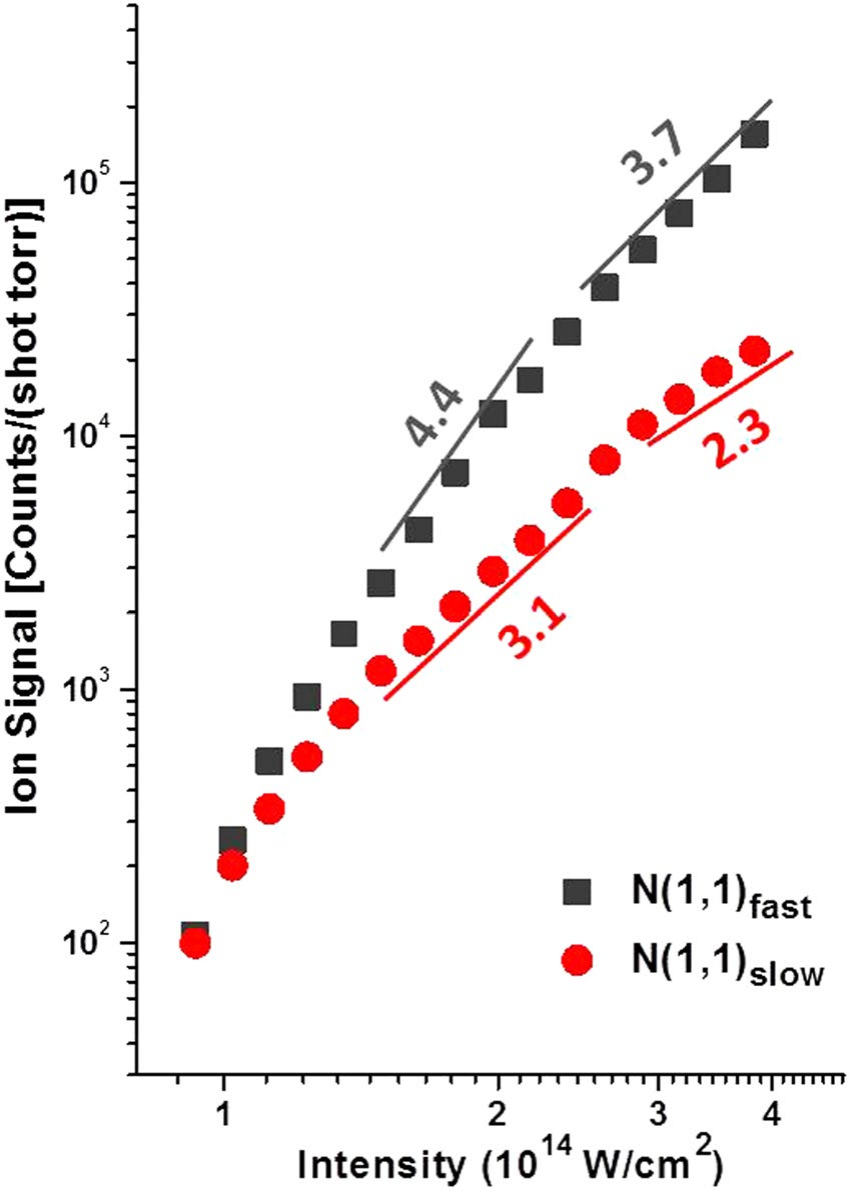
Fitting with the exponential order of the ion yield curves for N(1,1)_fast_ and N(1,1)_slow_ at the intensity corresponding to 2*I*_*0*_ and 4*I*_*0*_, where *I*_*0*_ = 10^14^ W/cm^2^.

Next, we compare the angular distribution data with the ionization rate of each channel obtained by ion yield curves^30, 31^. We plot the ion yield curves for N(1,1)_slow_ and N(1,1)_fast_ as a function of laser intensity in log scale in Fig. 4. The ion yield curves are obtained with the same TOF spectrometer using linearly polarized laser beam with polarization parallel to the molecular axis. By fitting the ion yield curves of N(1,1)_fast_ with an exponential function, we find that the exponential order is 3.7 and 4.4 at the intensities of 4*I*_*0*_ and 2*I*_*0*_, respectively, as marked in Fig. 4. The exponential order for N(1,1)_slow_ is 2.3 and 3.1 at the high and low intensities, respectively. Note that the exponential order is smaller at the high intensity end than the low intensity end for both channels and this is due to saturation in these channels and/or depletion of this channel by higher charged states when intensity gets higher^22, 31^.

The fitting order of the angular distribution data at the high and low intensities, 3 and 4, coincides with the exponential order of the ion yields of N(1,1)_fast_, 3.7 and 4.4, at the corresponding intensities, as shown in Fig. 4. The small discrepancy could be due to the contribution from that the perpendicular component of the laser field with respect to the molecular axis. However, this component should only play a minor role since we see a very weak signal strength of N(1,1)_fast_ when the laser polarization is perpendicular to the molecular axis (i.e. the horizontal component in Fig. 2). On the other hand, the consistence between the fitting of the angular distribution data and the ion yield curves of N(1,1)_fast_ indicates that the angular distribution of N(1,1)_fast_ is dominantly determined by its ionization rate, or more precisely, angular ionization rate, throughout our intensity range.

In contrast, the angular distribution of N(1,1)_slow_ is fitted by (*cos* ^2^ *θ*)^6^ at the high intensity end and (*cos* ^2^ *θ*)^5^ at the low intensity end, as shown in Fig. 3(c) and (d). The exponential fitting orders, 6 and 5, significantly diverge from the corresponding fitting orders of the ion yield curve, 2.3 and 3.1, as seen in Fig. 4. This indicates that the angular distribution profile of N(1,1)_slow_ is not solely determined by its angular ionization rate; instead, there must be other mechanisms leading to the substantial divergence between the angular distribution fitting and the ionization rate fitting, which very likely is the molecular alignment effect. The fact that the fitting order of the angular distribution profile is greatly larger than the ionization rate indicates that the molecules (or the fragmented ions) are more aligned towards the laser polarization when N(1,1)_slow_ is formed, resulting in a contracted distribution profile^5, 6, 11, 26^. Furthermore, when the intensity increases from 2*I*_*0*_ to 4*I*_*0*_, the fitting order of ionization rate of N(1,1)_slow_ decreases from 3.1 to 2.3. However, the fitting order of the angular distribution profile increases from 5 to 6. The greater discrepancy between the two fittings as intensity increases indicates that the alignment degree in N(1,1)_slow_ is increased when intensity increases.

### Formation dynamics of the degenerated dissociation channels

To understand why we observe different alignment effect in these two degenerated channels, we take a look at their formation dynamics. Our previous studies have shown that N(1,1)_fast_ involves a nonsequential double ionization (NSDI) transition, where the two electrons are removed almost simultaneously when the laser intensity rises sufficiently high^25, 32, 33^. In contrast, N(1,1)_slow_ is formed through a sequential double ionization (SDI) via enhanced ionization (EI)^23^ in a two-step transition: N_2_ firstly loses one electron and starts to dissociate, followed by an enhanced ionization of a second electron when the internuclear distance reaches the critical internuclear distance *R*_*c*_ in about 43 fs^23, 34^. Therefore, N(1,1)_fast_ is mostly formed at the leading edge of the 68-fs pulse, while N(1,1)_slow_ is formed at the trailing edge. This is further confirmed by a time sequence study with varying pulse durations as shown in Fig. 1(a) and (b). Figure 1(a) shows the TOF spectrum of N^+^ obtained with a longer 68-fs pulse and 4(b) with a shorter 45-fs pulse. We can see that the N(1,1)_slow_ peaks nearly disappear in the shorter 45-fs pulses and that is because the pulse duration is insufficient for this channel to form; in contrast, N(1,1)_fast_ consistently presents in both 45 and 68-fs pulses, indicating that this channel is formed at the pulse leading edge. A schematic of the formation time sequence of N(1,1)_slow_ and N(1,1)_fast_ is shown in Fig. 1(c).

## Discussion

Previous studies have shown that molecular alignment can be achieved in as fast as tens of femtoseconds for small molecules exposed to intense laser fields^5, 7, 8^. For example, when N_2_ is exposed in 45-fs pulse with a peak intensity of 1.4 × 10^14^ W/cm^2^, the highest degree of alignment happens at 67 fs follows the aligning pulse^8^. If an event happens in a similar time scale, it will strongly experience the molecular alignment effect. Since the SDI channel N(1,1)_slow_ is formed at the trailing edge of the laser pulse in about tens of fs^23, 34^, similar to the alignment time scale of N_2_, N(1,1)_slow_ will strongly experience an alignment effect (and possibly some post ionization alignment effect^12^), i.e. the molecules (or molecular ions) that form N(1,1)_slow_ experience stronger alignment and therefore a higher degree of alignment is seen in its angular distribution. As the laser intensity increases, laser field-induced alignment becomes stronger^5, 6, 8, 9, 11, 35^, and this leads to the contraction as we see in the angular distribution of N(1,1)_slow_ in Fig. 2.

In contrast, the NSDI channel N(1,1)_fast_ is formed at the leading edge of the laser pulse, where both electrons are almost simultaneously removed as fast as the laser intensity rises sufficiently high^25, 32, 33^, followed by strong Coulomb explosion and molecular bond breaking. Therefore, the NSDI channel only experiences limited alignment effect and/or post ionization alignment^12^. As a result, the angular distribution of N(1,1)_fast_ is less affected by molecular alignment effect, instead, is dominantly determined by its intensity-dependent ionization rate, which leads to the coincidental fitting between the angular ion distribution data and the ionization rate curve as discussed above.

Note that in our current experimental setup we could not exclude the possibility of the post-ionization alignment effect contributing to our experimental results besides the molecular alignment effect. Some thoughts to investigate the relative strength of the post-ionization alignment effect in the SDI channel N(1,1)_slow_ are provided here for future studies: a pump-probe experiment consisting of pump pulse duration being long enough for molecular alignment effect to take place but less than the critical time to trigger the SDI N(1,1)_slow_ channel (e.g. 45 fs, which was reported to well align N_2_ molecules [8] but insufficient to produce N(1,1)_slow_ [23]), and probe pulse duration being much shorter (e.g. a few fs) to trigger the SDI N(1,1)_slow_ channel at the critical nuclear distance, *R*_*c*_. The angular distribution of the SDI N(1,1)_slow_ channel from this pump-probe experiment, if compared with single- long-pulse experiment results (e.g. the results shown in this paper), could reveal the relative strength of the post-ionization alignment effect. Here we note on the relative strength and that is because the parental state of the N(1,1)_slow_ channel, i.e. the N_2_^+^ state, may involve both the molecular alignment and the post-ionization alignment effects since single ionization of N_2_ could happen immediately once the pump pulse arrives and therefore it may not be easy to completely exclude post-ionization alignment effect in the N_2_^+^ state.

In summary, we perform a study on the strong-field molecular alignment effect in two degenerated channels from double ionization-induced dissociation of N_2_. Our previous study identified two N^+^ + N^+^ states, a fast channel with a higher KER and a slow channel with a lower KER; the fast channel is a nonsequential channel and the slow is a sequential channel. By measuring the angular distribution of dissociation fragments, we observe opposite angular distribution development in these two channels as the laser intensity increases, one expanding and one contracting. Our further study shows that the expanding angular distribution comes from the nonsequential channel, whereas the contracting angular distribution comes from the sequential channel. A further analysis of the time sequence of the sequential and nonsequential transitions reveals that the opposite angular distribution development is due to different degrees of strong-field induced molecular alignment in these two degenerated channels.

## Methods

Ion detection and collection is realized with a recently modified time-of-flight (TOF) spectrometer^22, 23^. The chamber base pressure is less than 5.0 × 10^−10 ^Torr. We measure the molecular orientation distribution by using our TOF spectrometer. The 2.5-mm pinhole opening on the voltage plates in our TOF spectrometer ensures that only ions having their velocity aligned with the TOF axis will be detected. A half-wave plate (HWP) is used before the focusing lens to control the angle of the laser polarization with respect to the TOF axis. Ion signal of a specific channel is collected as we rotate the HWP, which is equivalent to a scanning of the angular distribution of this channel. Figure 2(a) illustrates the geometric configuration of the experimental setup.

The laser used is a Ti:sapphire system consisting of a regenerative amplifier and a multi-pass amplifier that delivers pulses of 1.0–1.2 mJ/pulse at a 1 kHz repetition rate with the central wavelength at 800 nm. By adjusting the bandwidth of the seed pulse, 25 and 50 nm used in this work, before entering the regenerative amplifier, we manage to generate two pulse durations at 45 and 68 fs, respectively. To minimize the chirp of both pulses, we carefully tune the stretcher and compressor while monitoring the second and third order dispersion with a home-built FROG-like spectrum and phase detection system. This approach allows us to minimize the second and third order dispersion (higher order dispersion may still exist) and therefore achieve the closest-to transform-limited pulses.
